# Simultaneous removal of concentrated organics, nitrogen and phosphorus nutrients by an oxygen-limited membrane bioreactor

**DOI:** 10.1371/journal.pone.0202179

**Published:** 2018-08-30

**Authors:** Shengyun Yang, Gang Yao

**Affiliations:** College of Architecture and Environment, Sichuan University, Chengdu, China; University of Missouri, UNITED STATES

## Abstract

Simultaneous removal of organics, nitrogen and phosphorus was achieved in a bench-scale oxygen-limited membrane bioreactor (OLMBR). Due to the limited dissolved oxygen (~ 0.2 mg/L equilibrium concentration) and the increased sludge concentration associated with the hollow fiber membrane, the OLMBR was endowed with an excellent performance on the removal of multi-pollutants. The optimized removal efficiencies of COD, nitrogen (N), and total phosphorus (TP) were approximately 95.5%, 90.0% and 82.6%, respectively (COD/N/P = 500:10:1, influent loading = 5.0 kg COD·m^-3^·d^-1^, 35°C). Mass balance and bacterial community analysis indicated that the removal of organic carbon was mainly achieved by the methane production process (67.6%). Short-cut nitrification-denitrification (SCND) was observed as the primary denitrification process in the OLMBR, in which the concentrated organic compounds served as the electron donors for denitrification. Nitrosomonas was observed to be the predominant ammonium-oxidizing bacteria, while nitrite-oxidizing bacteria were almost absent in the microbial community as revealed by the high-throughput sequencing technique. In addition, *Euryarchaeota* and *Candidatus*, which were well associated with the process of denitrifying anaerobic methane oxidation, were also detected. Sludge absorption was the main route for TP removal in the OLMBR, and the production of PH_3_ gas also accounted for 19.4% of TP removal. This study suggested that the interception effect of hollow fiber membrane provided higher sludge concentration, therefore offering more bacteria for pollutant removal. The OLMBR can be used for simultaneous removal of highly concentrated organics and nutrients in livestock and poultry breeding wastewater.

## Introduction

Livestock and poultry breeding wastewater usually has high concentration of COD, nitrogen and phosphorous, and its discharge may pose great harm to local water environment [1]. Physical and chemical methods, such as adsorption and chemical reaction, could achieve the removal of these pollutants, however, they often resulted in high cost [2]. Treating the wastewater via aerobic biochemical reaction has the advantages of high reaction rate and improved removal efficiency. However, it usually requires to maintain a concentration of dissolved oxygen (DO) higher than 2 mg/L in the wastewater, leading to a higher energy consumption. In addition, the removal of phosphorous is also difficult by a simple aerobic process. Alternatively, the use of anaerobic biochemical process to remove the aqueous contaminants could produce methane (CH_4_) for energy utilization and the operating cost is relatively low. However, the anaerobic biochemical process also faces the problem that the reaction rate is low and the effluent requires further treatment to reach the standard of wastewater discharge.

In recent years, membrane bioreactor (MBR) technology has been widely studied and applied in the treatment of municipal and industrial wastewater [3–5], including aerobic MBR, anaerobic MBR and facultative MBR. MBR technology can offer particle-free high-quality effluent and withstand high organic loading conditions [6,7]. In order to keep oxic environment, higher energy consumption is needed for an aerobic MBR technology [8]. Anaerobic MBR technology is suitable for the treatment of concentrated wastewater, producing CH_4_ for energy utilization but less sludge. This technology has been extensively studied to improve the COD removal efficiency of wastewater [9,10]. Using a submerged anaerobic MBR for urban wastewater treatment reduces sludge production, eliminates aeration and generates methane, meanwhile, anaerobic MBR allows meeting longer sludge retention times, which is the main requirement necessary for high-rate anaerobic treatment [11]. However, an anaerobic MBR-based technology does not work for the nutrient removal of wastewater, so it is not suitable for the water treatment process when downstream treatment or alternative water reuse (e. g. irrigation) are not considered [12].

In order to develop an energy-saving membrane-assisted bioreactor capable of treating COD and nutrients simultaneously, facultative MBR was proposed and investigated by some scholars [13–15]. For a facultative bioreactor, the oxygen-limited environment may allow the coexistence of nitrifying bacteria and denitrifying bacteria, thereby achieving a simultaneous nitrification and denitrification (SND) process[14–17]. Therefore, facultative MBR is expected to combine the advantages of membrane-based bioreactor and the oxygen-limited environment for maximizing nutrient removal. The enrichment of various functional bacteria by hollow fiber membrane could achieve the removal of multi-pollutants. With the co-existence of anaerobic area, anoxic area and aerobic area, the facultative MBR may provide the possibility of achieving a simultaneous removal of COD, nitrogen and phosphorus in wastewater. However, the previous research work did not give more information regarding how nutrients were simultaneously removed in membrane-based bioreactor under an oxygen-limited condition; moreover, a precise control on the oxygen concentration was never adopted in the previously reported works.

In this study, we propose an oxygen-limited membrane bioreactor (OLMBR) for the treatment of wastewater containing concentrated organics and nutrients (N and P). In contrast to the previously reported facultative MBR, we deliberately control a DO equilibrium concentration of ~0.2 mg/L in the OLMBR, and found that this DO level was an optimal value offering efficient removal of COD, N and P in the OLMBR. The mass balance of C, N and P in the system was calculated based on the experimental data, and gene testing was employed for analyzing bacterial community of the biofilm on hollow fiber membrane to reveal the biological mechanism of nutrient removal. The OLMBR demonstrated in this study may provide clues for developing a compact water facility to cope with the wastewater containing concentrated organics, nitrogen and phosphorous.

## Materials and methods

### Wastewater composition and seed sludge

The wastewater in this work was synthesized with glucose, KH_2_PO_4_ and NH_4_Cl as the carbon source, phosphorus source and nitrogen source, respectively. Unless otherwise noted, the ratio of COD/N/P in the simulated wastewater was fixed at 500/10/1 and the inlet flow was controlled at 50 L/d. Na_2_CO_3_ and NaHCO_3_ were used to keep the pH value in the reactor at 7.0–7.8. In addition, to obtain a balance feed for microbial growth, some trace elements were introduced to the wastewater. The seed sludge was obtained from the secondary clarifier in the Zhaoyang municipal wastewater treatment plant (28.650^o^ Lat./N-115.867^o^ Lon./E, Nanchang, China). The collection of seed sludge was permitted by the technical manager of the plant.

### Experimental setup

**Fig 1:**
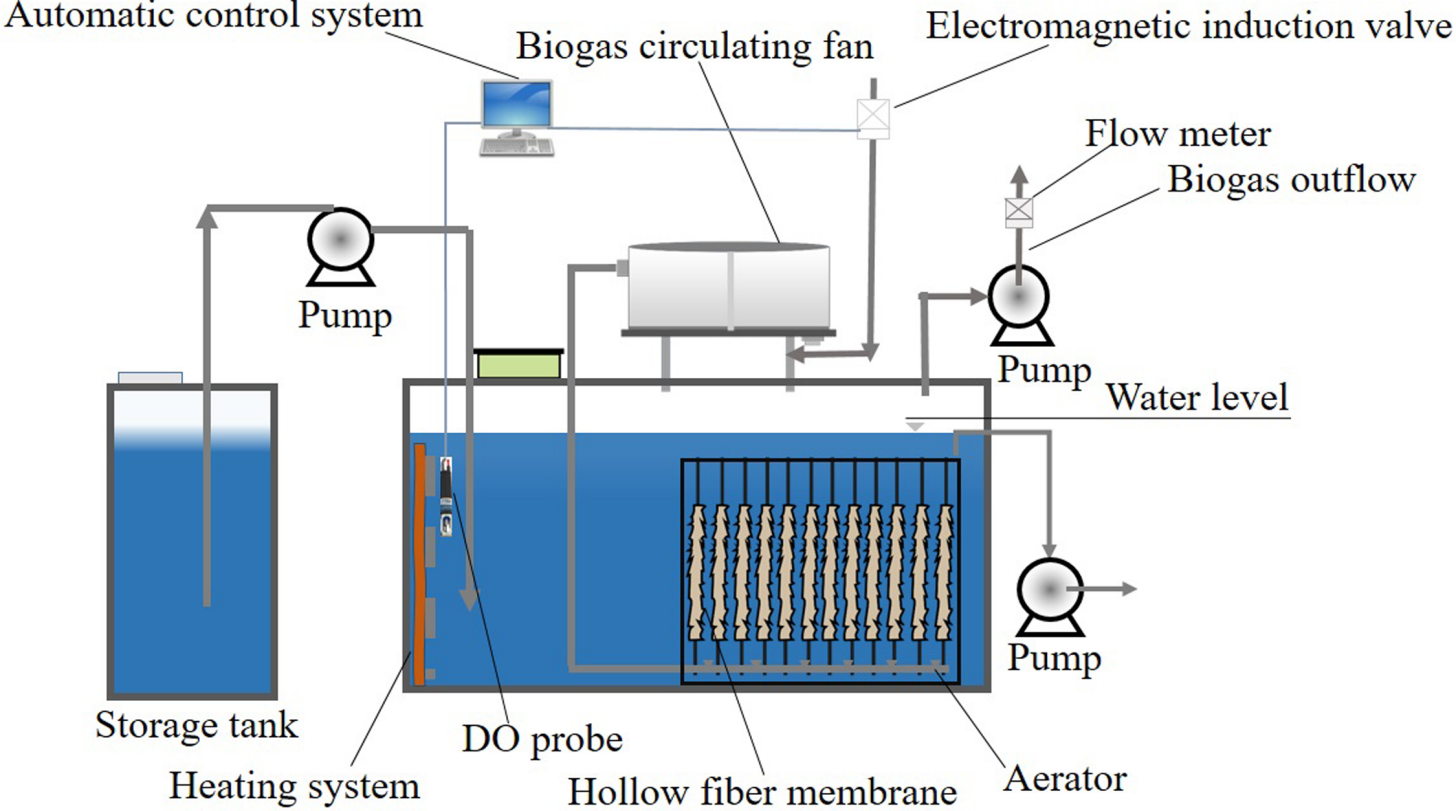
Illustration of the bench-scale oxygen-limited membrane bioreactor (OLMBR) in this study.

As shown in Fig 1, experiments were carried out in a bench-scale oxygen limited membrane bioreactor made of polyvinyl chloride (PVC) with working volume of 0.1 m^3^. The temperature was controlled by an aquarium heater. The micro-pore aeration device was installed at the bottom of the reactor. In order to control the DO equilibrium concentration (e. g. ~0.2 mg/L in this study) after the initial DO dropping stage (~ 6 days), the signal of the DO probe was transferred to the computer to control the air gas automatic valve. When the DO concentration was below 0.15 mg/L, the solenoid valve opened automatically and allowed air to enter into the reactor. This automatic system controlled the concentration of dissolved oxygen at about 0.2 mg/L. The inlet air was diluted by the recirculated gases production to obtain methane and a limited concentration of dissolved oxygen. The hollow fiber membrane made of polyvinylidene fluoride (PVDF) was used as the membrane material in the reactor. The membrane unit was immersed under the water with 0.4 m^2^ area. The temperature was controlled at 35°was conducted based on the StandardC during the initiation stage. If not elsewise specified, the COD concentration of influent was kept at ~5000 mg/L, and the flow rate was 50 L/day. For the start-up stage, tap water was prefilled in the bioreactor. At the start-up stage, the removal efficiency of COD and NH_4_^+^-N increased gradually and reached a steady state finally. Then the effects of operating parameters on the removal of COD, N and P were studied individually. Before the study of an individual factor, a standard influent condition (COD/N/P = 500:10:1, influent loading = 5.0 kg COD·m^-3^·d^-1^, 35°C) was used at least for one week, and the removal efficiencies of COD/N/P were evaluated to ensure that the bioreactor was working at a same steady stage. The whole experimental period of OLMBR was 15 months, and the water flux of membrane stabilized at ~5 L/m^2^·h. The sludge were discharged for two times (the 7^th^ month and 11^th^ month) over the whole experimental period to maintain a suspended sludge concentration lower than 15 g/L. At the same time, the hollow fiber membrane were taken out for a cleaning operation (washing with 3% sodium hypochlorite).

### Analytical methods

#### Sample and data analysis

The analysis of COD, TN, NH_4_^+^-N and NO_3_^-^-N in the system was conducted based on the Standard Methods for the Examination of Water and Wastewater[18]. For the detection of low concentration of NO_2_^-^-N and NO_3_^-^-N, ion chromatography (IC) was used (Dionex ICS3000 equipment and IonPac AS19 analytic column). The gas compositions (CH_4_, H_2_, CO_2_, O_2_ and N_2_) in influent and effluent were examined by using gas chromatograph (GC-TCD, Micro GC 490, America). The elemental analysis (C, H, O, N) of the sludge samples were carried out on the elemental analyzer (VARIO MICRO, Germany). The qualitative detection of PH_3_ was examined by the gas detecting tube. For the further quantitative detection of PH_3_ content, the mixed gas was absorbed by NaOH solution and analyzed by the Mo-Sb colorimetric method. A pH meter (pHS-3C, Leici, China) was used to monitor the pH in the system.

#### Microbial community analysis

The technique high-throughput Ion Personal Genome Machine^TM^ (PGM^TM^) System sequencing (Thermo Fisher Scientific) of the 16rDNA was used to study the evolution of biological community of the OLMBR. The biofilms on the hollow fiber membrane after domestication were sampled by repeatedly scraping the surface of fiber using a sterile blade. The samples including original seed sludge and scraped biofilm were stored at -20°C until analysis[19]. The DNA was extracted from 500 mg sample using the MoBio PowerSoil DNA extraction kit (MOBIO Laboratories, Loker Ave West, Carlsbad, CA, USA) following the manufacturer’s instruction. The V4 region of bacteriall 16s RNA genes were amplified using the universal primers 515F (TCTATGTGCCAGCMGCCGCGGTAA) and 926R (CCGTCAATTCM TTTRAGTTT), in order to obtain a good coverage of almost all phyla in conventional and metagenomic studies [20–23].

Samples of gene were extracted for polymerase chain reaction (PCR) reactions according to the manufacturer’s instructions and previous reports [19,20]. Prior to library pooling, the barcoded PCR products were purified and quantified using the Qubits DNA HS Assay Kit (Life technologies, Carlsbad, CA, USA). The libraries were sequenced by 300bp on the MiSeq platform using MiSeq v3 Reagent Kit (Illumina). Sequence preprocessing was performed mainly upon software of mothur (version 1.35.1) and then aligned with the SILVA databases, version 119.

#### Mass balance calculation

**Fig 2:**
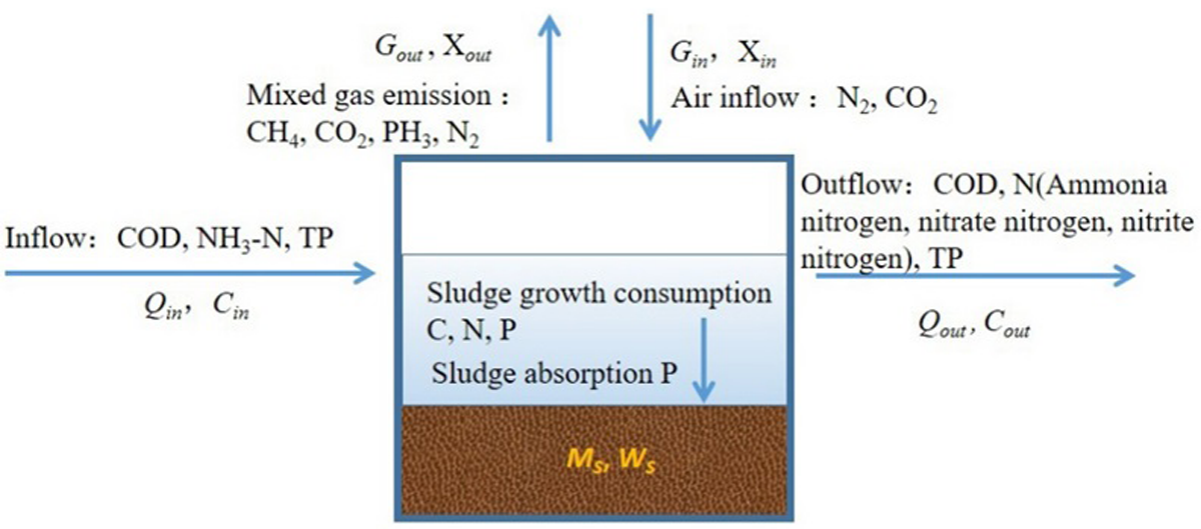
Mass balance diagram in the OLMBR. *Q*_*in*_*—*flow of influent (L/day), *Q*_*out*_*—*flow of effluent (L/day); *C*_*in*_*—*concentration of influent (mg/L), *C*_*out*_*—*concentration of effluent (mg/L); *G*_*out*_*—*volume of the daily emitted gas (L/d), *X*_*out*_*—*volume proportion of the emitted gas (%); *G*_*in*_*—*volume of the intake gas daily (L/d), *X*_*in*_*—*volume proportion of intake gas (%); *M*_*s*_*—*mass of the daily sludge increment (g/d), *W*_*s*_—element percentage (%).

The mass balance diagram is shown in Fig 2, and the experimental data are applied to the mass balance equations shown in Eqs (1)–(18). For convenience of calculation, the volume of the gas is converted to the volume under the standard condition. One mole of gas is 22.4 liters at the standard atmospheric pressure.

For calculation of the mass balance of carbon, COD is converted to glucose (C_6_H_12_O_6_). 1.06 g COD corresponds to 1g glucose (C_6_H_12_O_6_), which contains a carbon content of 40%.

$$M_{in(C)}=M_{gout(C)}+Ms_{\left(C\right)}+M_{out(C)}-M_{gin(C)}$$ (1)

$$M_{in(C)}=\sum (Q_{in(COD)}\times C_{in(COD)}÷1.06\times 40\%)$$ (2)

$$M_{gout(C)}=\sum (G_{out}\times X_{out(CO_{2}}{}_{)}+G_{out}\times X_{out(CH}{}_{{}_{4})})÷22.4\times 12$$ (3)

$$M_{gin(C)}=\sum (G_{in}\times X_{in(CO}{}_{{}_{2})})÷22.4\times 12$$ (4)

$$M_{S(C)}=\sum (M_{S}\times W_{S(C)})$$ (5)

$$M_{out(C)}=\sum \left(Q_{out(COD)}\times C_{out(COD)}÷1.06\times 40\%\right)$$ (6)

Where *M*_*in(C)*_ and *M*_*out(C)*_ are total carbon in the influent and outflow of the reactor, respectively. *M*_*gin(C)*_ and *M*_*gout(C)*_ represent the inflowing carbon and outflowing carbon in the form of gas. *Q*_*in(COD)*_ and Q_out(C*OD*_) represent the inflow and outflow rate of the wastewater, while *C*_*in(COD)*_ *and C*_*out(COD)*_ represent the concentration of COD. For the intake gas, the inflowing CO_2_ from atmosphere is calculated as the input carbon source (Eq 4). For the outflowing gas, both emitted CO_2_ and CH_4_ were detected and analyzed, and no other volatile organic gases were considered. *M*_*S(C)*_ represents change of the carbon mass associated with the sludge growth, and *W*_*S(C)*_ is the carbon element percentage of the mass increment of sludge. − is the summation of the targeting parameter in the duration of experimenting time for mass balance (24 days in this study).

$$M_{in(N)}=M_{s(N)}+M_{out(}{}_{NH_{3}-N)}+M_{out(}{}_{NO_{3}\;-N)}+M_{out(}{}_{NO_{2}\;-N)}+Mg_{\left(N\right)}$$ (7)

$$M_{in\left(N\right)=}M_{in(NH_{3}-N)}=\sum \left(Q_{in(NH_{3}-N)}\times C_{in(NH_{3}-N)}\right)$$ (8)

$$Mg_{\left(N\right)}=\sum (G_{out}\times X_{out(N}{}_{{}_{2})}-G_{in}\times X_{in(N}{}_{{}_{2})})÷22.4\times 28$$ (9)

$$M_{S(N)}=\sum (M_{s}\times W_{S(N)})$$ (10)

$$M_{out(}{}_{NH_{3}-N)}=\sum (Q_{out}\times C_{out(}{}_{NH_{3}-N)})$$ (11)

$$M_{out(}{}_{NO_{3}\;-N)}=\sum (Q_{out}\times C_{out(}{}_{NO_{3}\;-N)})$$ (12)

$$M_{out(}{}_{NO_{2}\;-N)}=\sum (Q_{out}\times C_{out(}{}_{NO_{2}\;-N)})$$ (13)

Eqs (7)–(13) show the methodology for the calculation of the mass balance of nitrogen, where M_in(N)_ is total nitrogen(TN) in the influent, while *Mg*_*(N)*_ represents nitrogen gas (N_2_) produced by denitrification. *M*_*S(N)*_ represents the mass change of N element associated with the sludge growth and *W*_*S(N)*_ is the element percentage of N. *M*_*out(NH_3_-N)*_, *M*_*out(NO_3_ -N)*_ and *M*_*out(NO_2_ -N)*_ are the mass of NH_3_-N, NO_3_-N and NO_2_-N in the effluent, while *C*_*out(NH_3_-N)*_, *C*_*out(NO_3_ -N)*_ and *C*_*out(NO_2_ -N)*_ represent the concentrations of NH_3_-N, NO_3_-N and NO_2_-N in the effluent, and *Q*_*out*_ is the outflow rates, respectively.

$$M_{in(P)}=M_{s(P)}+M_{out(P)}+Mg_{\left(P\right)}$$ (14)

$$M_{in}=\sum \left(Q_{in(P)}\times C_{in(P)}\right)$$ (15)

$$Mg_{\left(P\right)}=\sum (G_{out}\times X_{out(PH}{}_{{}_{3})})÷22.4\times 31$$ (16)

$$M_{S(P)}=(M_{s}\times W_{S(P)})$$ (17)

$$M_{out(P)}=\sum \left(Q_{out(P)}\times C_{out(P)}\right)$$ (18)

For the calculation of the mass balance phosphorus, Eqs (14)–(18) are used, where M_in(P)_ and Mg_(P)_ represent total phosphorus in the influent and effluent, respectively. Mg_(P)_ and M_S(P)_ represent the phosphine in the outflow and the phosphorus consumption caused by sludge growth, respectively.

## Results and discussion

### Performance of OLMBR at start-up stage

#### DO at start-up stage

**Fig 3:**
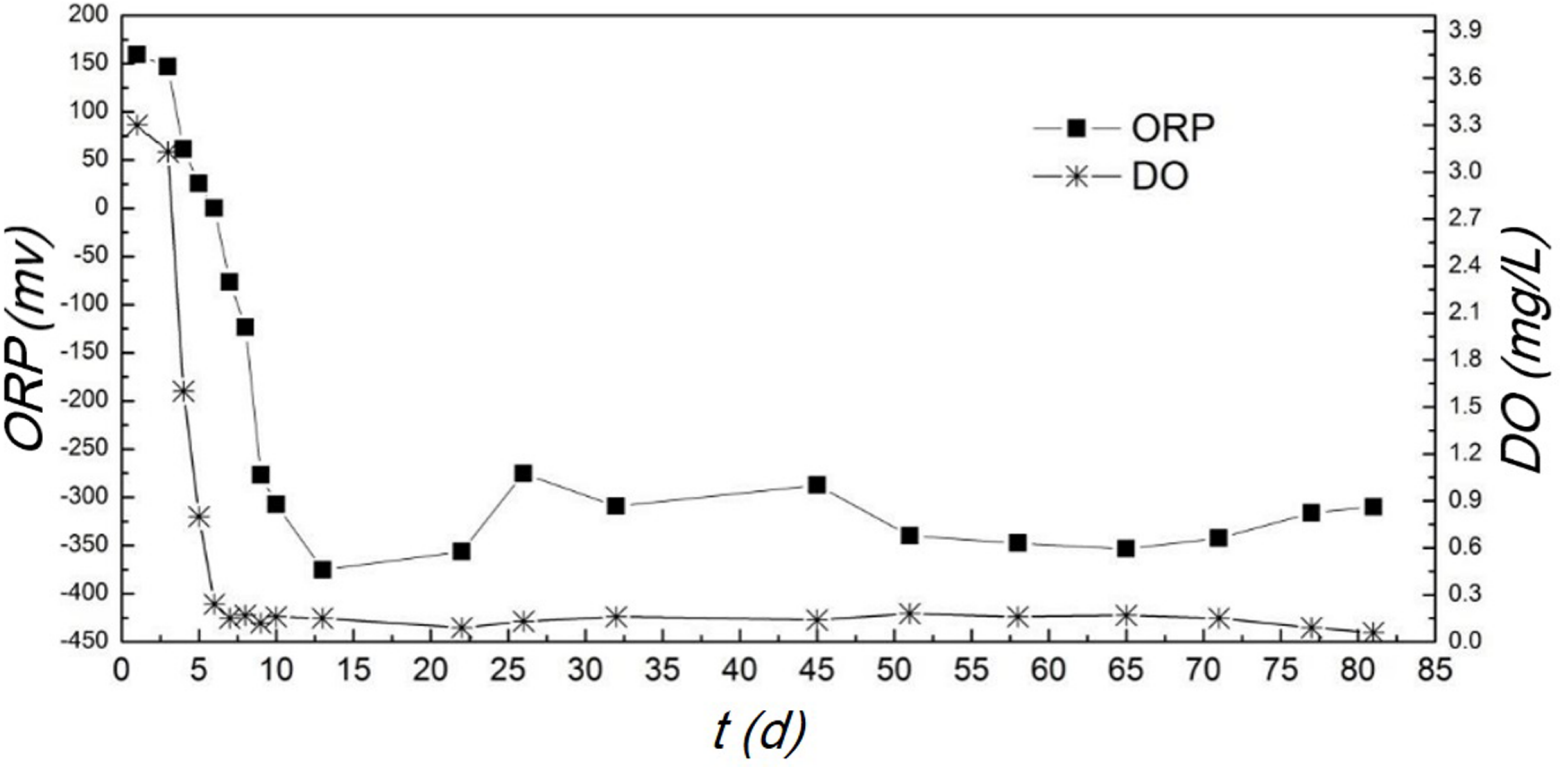
DO and ORP profiles for the start-up stage of the OLMBR.

DO concentration is essential to a bioreactor for the removal of COD, N and P[24]. The DO concentration and oxidation-reduction potential (ORP) in the OLMBR was continuously measured and the results are displayed in Fig 3. It can be seen that the DO in the system significantly decreased on the third day and finally equilibrated at around 0.2 mg/L. The ORP at steady state was in the range of -250 to -350 mV. It indicates a stable status between anaerobic and anoxic conditions [25–26] formed in the system. With a high concentration of organic carbon source in the system, dissolved oxygen was quickly consumed in the initial stage. This observation suggests that facultative microorganism and anaerobic microorganism (methanogens) in the sludge finally became the dominant species and consequently the anaerobic biochemical reactions played a major role in the system. Overall, the DO in the system was kept around 0.2 mg/L and an oxygen-limited environment was successfully established with the assistance of the automatic valve for air inlet.

#### COD removal at start-up stage

**Fig 4:**
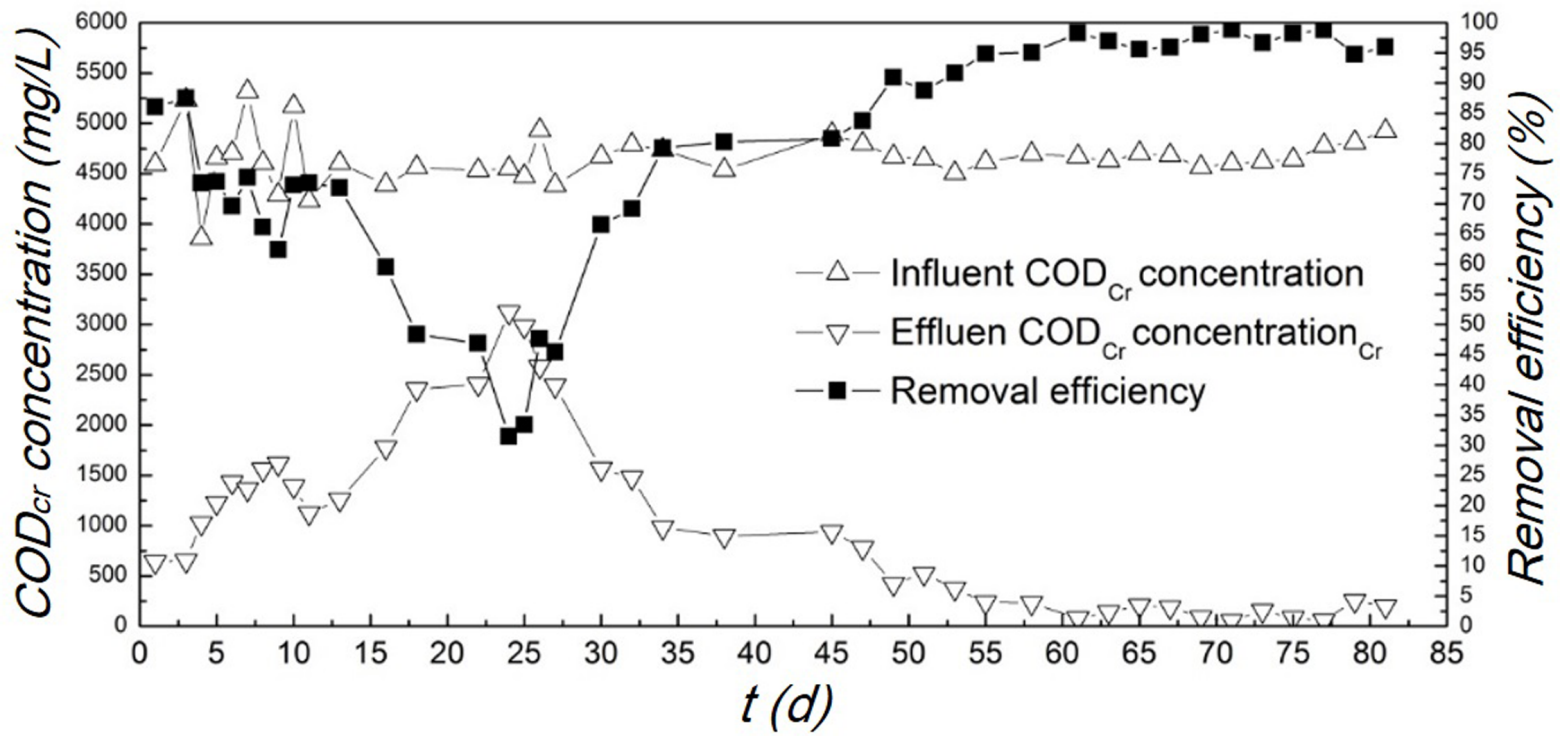
Profiles of the COD concentration and removal efficiency over the start-up stage.

As shown in Fig 4, a relatively low concentration of COD was soon obtained in the effluent during the initial 10 days, owing to that the influent was diluted by the prefilled tap water in the reactor. Afterwards the COD removal efficiency quickly decreased over the following 10 days due to the sluggish kinetics of the bio-process. On the 25^th^ day, the anaerobic and facultative microorganisms appeared to take effect and the COD removal efficiency was gradually improved. On the 55^th^ day, the reactor maintained a COD removal efficiency more than 95%.

On the 49^th^ day, the composition of gas produced was examined by gas chromatograph and the result was presented in S1 Table. The amount of CH_4_ accounted for 50.7% of the headspace gas, which indicated that CH_4_ could be generated continuously via the biochemical reactions in the system. CO_2_ was also detected and accounted for the 27.9% of the gas. Obviously, the presence of limited DO did not affect the enrichment and metabolic activity of the methanogenic bacteria. The presence of DO was usually unfavorable to the anaerobes. However, it was reported that methanogens could maintain high activity at low ORP in the presence of minute quantities of O_2_[27]. In an oxygen-limited reactor, the oxygen diffusion limitation contributed to the formation of anoxic micro-environment in the sludge. Anaerobic bacteria and facultative bacteria could coexist in the same reactor due to the different dissolved oxygen levels, and this is the important characteristic of the OLMBR.

#### NH_4_^+^-N removal at start-up stage

**Fig 5:**
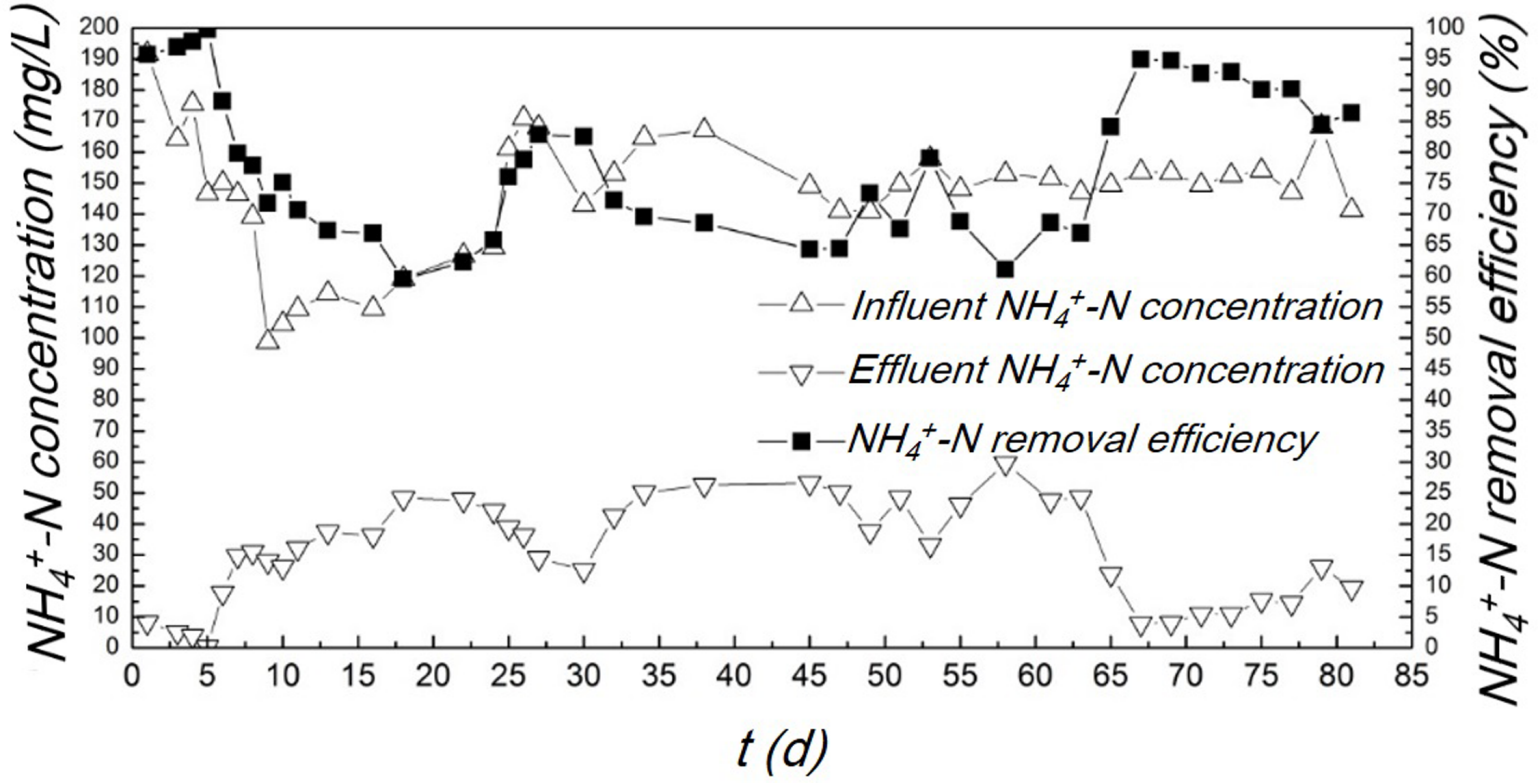
NH4^+^-N concentrations and removal efficiency over the start-up stage.

The NH_4_^+^-N removal during the start-up stage is displayed in Fig 5. It could be seen that the NH_4_^+^-N removal efficiency steadily increased and maintained above 80% when the influent loading of NH_4_^+^-N reached ~130 mg/L finally. Acid molecules came from the fermentation reaction made the pH in the system lower than 9.5. Thus, NH_4_^+^-N should exist as ionic state in the water. Moreover, the gas chromatograph results demonstrated that ammonia molecules were not detected in the mixed production gas. Therefore, the decrease of NH_4_^+^-N in the system should be attributed to the SND reaction, rather than the gas stripping process. Compared to the nitrite oxidizing bacteria, ammonia oxidizing bacteria have stronger adaptability to environment. The growth rate of nitrite oxidizing bacteria was reportedly lower than that of ammonia oxidizing bacteria at 35°C[15]. Under the low DO condition, ammonia oxidizing bacteria take the priority to survive and utilize the oxygen molecules, since ammonia oxidizing bacteria has strong affinity to oxygen[15, 28]. Therefore, the COD removal in the OLMBR reactor was mainly via the anaerobic process, while the ammonia removal was according to the SND reaction.

**Fig 6:**
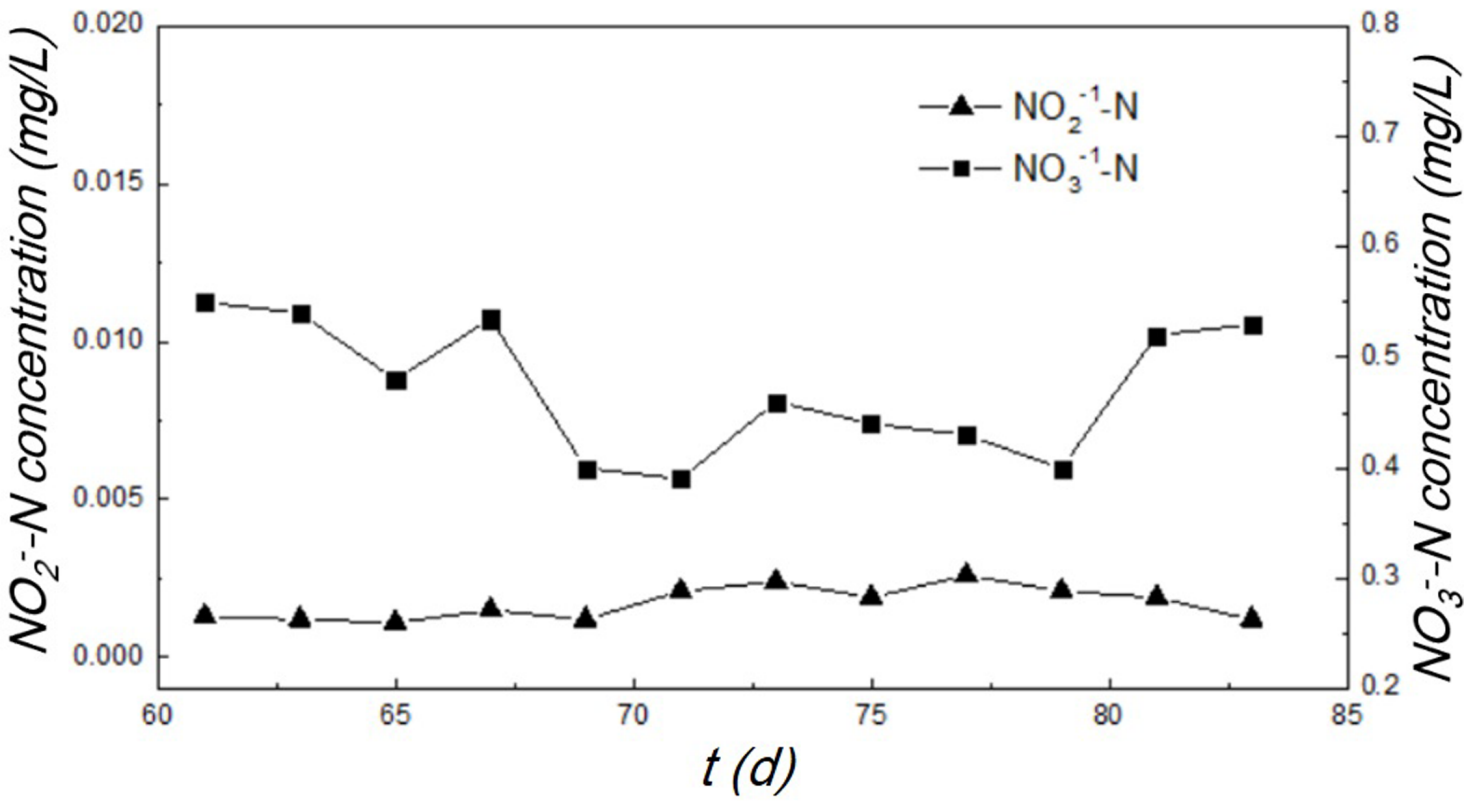
NO_2_^-1^-N and NO_3_^-1^-N concentration in effluent over the start-up stage.

The concentrations of NO_3_^-1^-N and NO_2_^-1^-N in the effluent are displayed in Fig 6. The NO_3_^-1^-N concentration in the effluent ranged from 0.38–0.56 mg/L. The NO_2_^-1^-N concentration was close to the detection limit (i.e. 0.001 mg/L for the IC analytical method used in this study). Since a high NH_4_^+^-N removal efficiency was achieved in the OLMBR, the presence of low level of NO_3_^-1^-N and NO_2_^-1^-N confirmed the occurrence of denitrification reaction. Under the limited DO condition, previous works proved that the aerated nitrification of ammonia nitrogen was primarily controlled at the nitrosation stage rather than the nitration stage in the bio-system [29], namely the so-called short-cut nitrification-denitrification (SCND). However, although a desirable ammonia removal efficiency was achieved in the OLMBR, the accumulation of nitrite was not observed since the NO_2_^-1^-N was only several micrograms per liter. This observation is different from the generally reported SCND processes, in which the higher levels of nitrite were often observed in the effluents [30–34]. We think this phenomenon is most likely associated with the enhanced microbial activity in the OLMBR bioreactor relative to that in the conventional anaerobic granular sludge. The sludge retention effect of hollow fiber membrane enabled sufficient sludge to treat the wastewater with concentrated pollutants [15]. According to our measurement on sludge concentration, the suspended sludge concentration increased to 11 g/L in the OLMBR at the end of start-up stage, which was much higher than the value that an aerobic activated sludge process can normally achieve (i. e. 3–4 g/L). In addition to the SCND process, the anaerobic methane oxidation could occur under the condition of circulating methane aeration according to pervious literature [30,35]. The methane oxidation route, which occurred at a low DO concentration (< 1mg/L) and high CH_4_ concentration, may also accelerate the reduction of nitrite/nitrate via the strong reductive CH_4_ [30,35], thus promoting the denitrification in the biosystem. Therefore, the denitrification reaction can proceed completely, and no accumulation of nitrite/nitrate was observed.

#### TP removal at start-up stage

**Fig 7:**
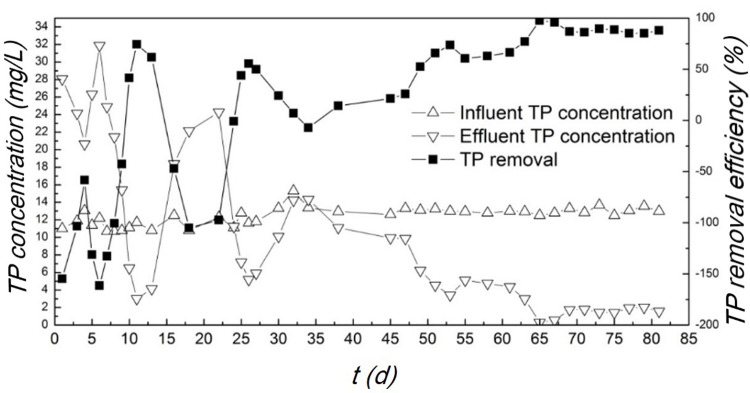
TP removal of the simulated wastewater over the start-up stage.

The TP removal at start-up stage is displayed in Fig 7. Over the first few days, it was observed that the TP concentration of effluent fluctuated and was even higher than that of influent. It is possibly due to the reason that the phosphorus adsorbed by polyphosphate accumulating bacteria or by the abiotic substances in sludge released to the water again. As time went by, the TP removal efficiency gradually increased and finally maintained at ~80%. This result indicates that the microbial flora that could give high phosphorus removal efficiency had been successfully cultivated under the oxygen-limited condition. Through the enrichment of various functional bacteria in the sludge and the establishment of the anoxic micro-environment in the membrane bioreactor, the TP removal by the phosphate anaerobic organisms could be achieved. According to reported research works, the reaction might be happened that the phosphorus accumulating organisms (PAOs) could use NO_3_^-^ and NO_2_^-^ to replace parts of O_2_ as the electron acceptor to degrade phosphorus-containing organics under the limited-oxygen condition, thereby absorbing phosphorus and promoting denitrification[36–38]. In addition to the PAOs, we also found another transformation route for the phosphorus. We examined the headspace gas and PH_3_ was detected (see S1 and S2 Figs. in the supporting information). The observation suggests that a part of phosphorus was removed in the form of phosphate gasification[39]. Multiple removal pathways of phosphorus co-existed in the system.

### Effect of operating parameters during steady stage

#### Effect of influent COD

**Fig 8:**
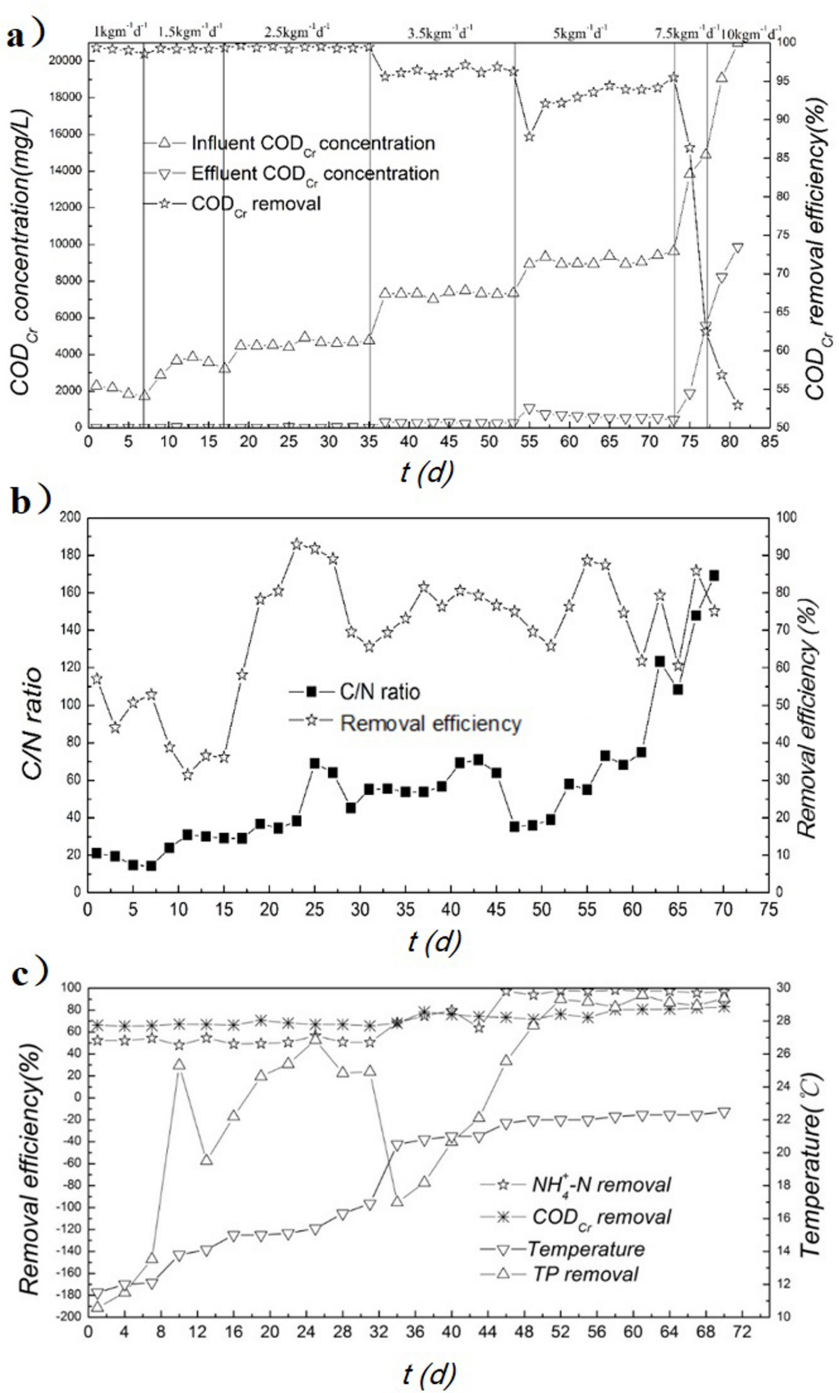
a) Effect of organic loading rate (OLR) on the removal efficiency of COD. b) Effect of C/N on the NH_4_^+^-N removal. Glucose was added into the system to achieve the different C/N ratios. The investigation was started with the condition of COD = 2000 mg/L and NH_4_^+^-N = 100 mg/L. c) Effect of temperature on the removal efficiency. The ratio of C/N/P in the simulated wastewater was fixed at 500/10/1 and the concentration of COD_Cr_ was about 5000mg/L.

In order to understand the characteristics of OLMBR, the effects of some operating parameters on the removal of pollutants during the steady stage were systematically investigated. The effect of COD concentration (influent loading) on COD removal is displayed in Fig 8A. It could be seen that a COD efficiency more than 90% could be obtained when the influent loading was lower than 5.0 kg·m^-3^·d^-1^. With the increase of influent COD concentration, the COD removal efficiency decreased gradually. This could be attributed to the acidification of the water. With the influent loading of 10.0 kg·m^-3^·d^-1^, the pH in the system was about 5.1. Low pH environment seriously restricted the activity of microbial system, thus leading to a poor COD removal efficiency. Therefore, the optimal influent loading was about 5.0 kg·m^-3^·d^-1^ under such conditions.

#### Effect of C/N on NH_4_^+^-N removal

The effect of C/N ratio on NH_4_^+^-N removal is displayed in Fig 8B. The results showed that the NH_4_^+^-N removal efficiency experienced a large fluctuation along with the change of C/N ratio. However, a basic rule can be observed that a higher C/N ratio offered a good performance on NH_4_^+^-N removal. When the COD concentration was low, carbon source was not enough to act as electron acceptors for denitrification reactions. Therefore, denitrification reactions were suppressed, leading to a lower NH_4_^+^-N removal efficiency. With more carbon sources in the system, the denitrification reactions were enhanced and it was beneficial to the NH_4_^+^-N removal. According to the investigation, the optimal C/N was about 50:1 for the OLMBR in terms of the removal of NH_4_^+^-N, and this value can guarantee a NH_4_^+^-N removal efficiency around 80%.

#### Effect of temperature

The effect of temperature on removal of pollutants is displayed in Fig 8C. It was observed that the temperature did not show an apparent effect on the removal of COD. As the temperature increased from 11°C to 23°C, the COD removal efficiency was around 70% all the time. When the temperature in the system was higher than 20°C, the NH_4_^+^-N removal efficiency increased by ~40% relative to the value achieved at 10°C. It was widely acknowledged that physiological activities of microorganisms would be suppressed at lower temperatures, resulting in the decrease of removal efficiency. However, at lower temperatures, a good performance on COD and NH_4_^+^-N removal was still obtained. This might be attributed to that the enrichment of various functional bacteria mitigated the inhibitive effect of lower temperatures on the removal of pollutants. The TP removal efficiency also increased to values more than 80% when the temperature reached 22°C, although large fluctuation was still found in the system. As aforementioned, the fluctuation is mainly associated with the release of phosphorous in sludge.

### Bacterial community analysis of the biomembrane

**Fig 9:**
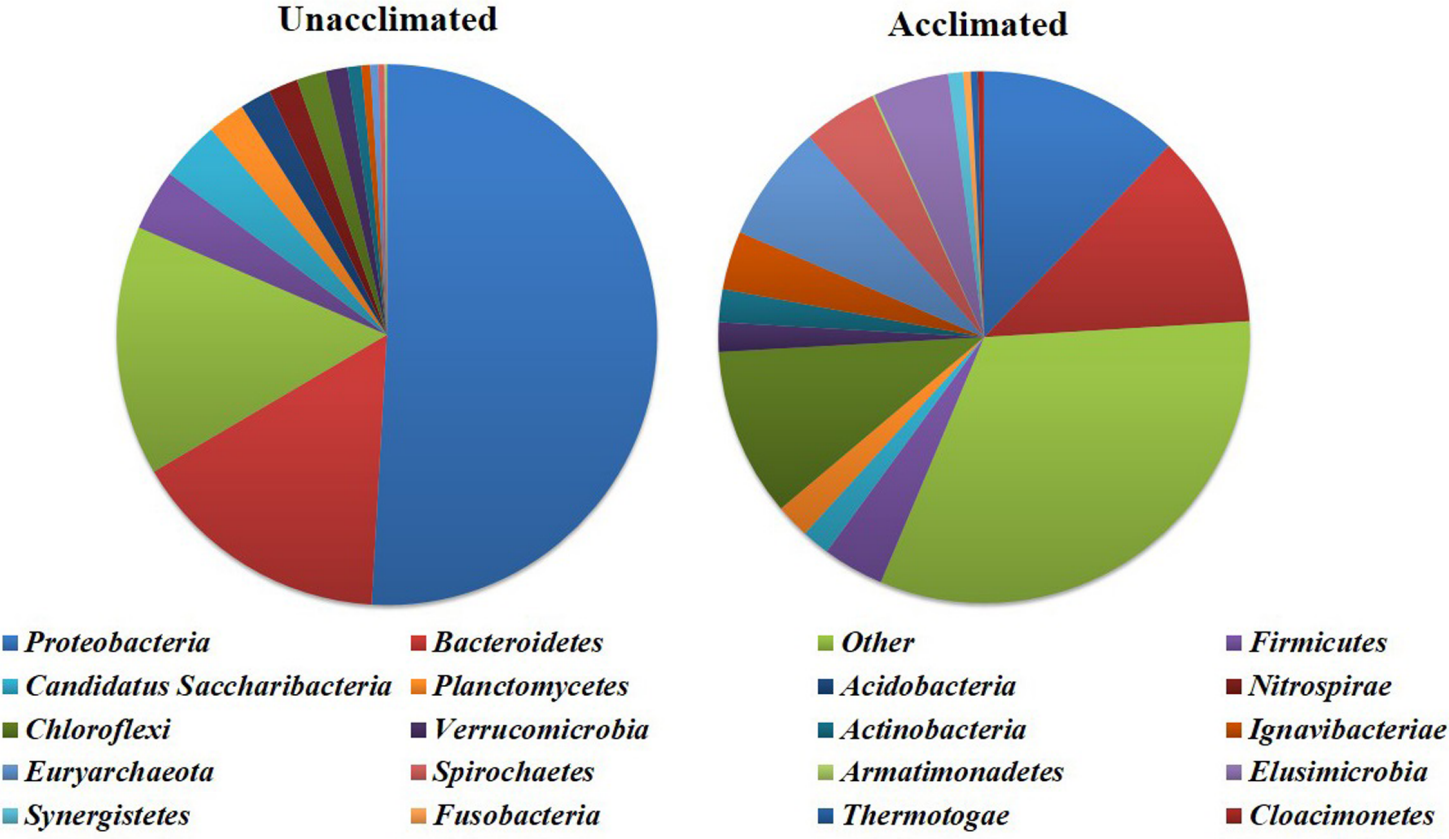
Illumina genome analyzer IIx result (Phylum level).

In order to interpret the biological mechanism of the removal of the pollutants, the difference of bacterial community between the original seed sludge (denoted as unacclimated) and the acclimated biofilms was investigated by high-throughput sequencing technique. The qualified reads were assigned with known phyla, classes, orders, Families and Genuses. As shown in Fig 9, *Proteobacteria* was the most abundant phylum before domestication and lower after domestication of 50.63% and 12.41%. The other dominant phyla included *Bacteroidetes*(15.56%), *Firmicutes*(3.63%), *Candidatus Saccharibacteria* (3.57%), *Planctomycetes*(2.23%) and *Chloroflexi*(1.74%) before domestication; and *Bacteroidetes* (11.98%), *Chloroflexi*(10.40%), *Euryarchaeota*(7.24%), *Elusimicrobia*(4.66%), *Spirochaetes*(4.57%), *Firmicutes*(3.79%) and *Ignavibacteriae*(3.61%) after domestication. Due to the environment change from aerobic to limited oxygen conditions, the bacterial community before and after domestication shared some differences in the predominant phylum, which were *Proteobacteria*, followed with *Bacteroidetes and Firmicutes*. The similar distribution of bacterial community in the process of acclimation has been detected in anaerobic reactor by previous scholars[40–44]. As the main anaerobic ammonia oxidizing bacteria, *Planctomycetes* exhibited the abundance of 2% before acclimation, while not detected after acclimation. This observation indicated that the denitrification by anaerobic ammonium oxidation was unavailable in the reactor[45–48]. The phyla of *Euryarchaeota* belonging to anaerobic bacteria increased greatly and then became a typical dominant species. In addition, the biofilms after domestication in this study seems to have more species and are more evenly distributed due to the oxygen limited condition.

**Fig 10:**
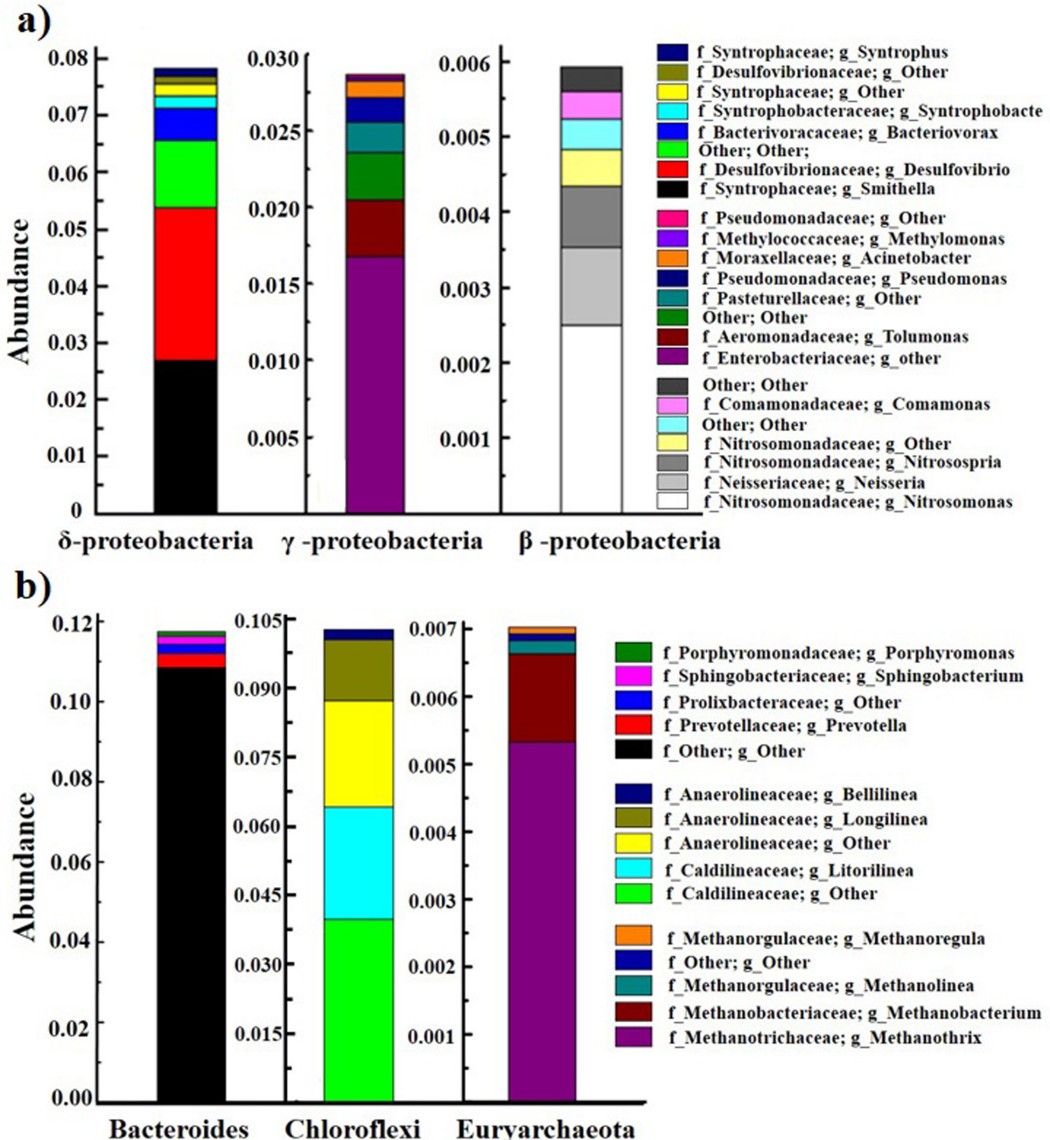
Genus level of bacteria for: a) *Proteobacteria*; b) *Bacteroidetes*, *Chloroflexi* and *Euryarchaeota*.

As shown Fig 10A, the most abundant phylum of the *Proteobacteria* were analyzed. In the phylum of microbial community distribution, the acclimated sludge groups were mainly *δ-proteobacteria* (8.29%) and *γ-proteobacteria* (2.87%), and only a small part of *β-proteobacteria* (0.59%). Smaller categories were further identified. The genuses of *Smithella*, *Desulfovibrio*, *Bacteriovorax* and *Syntrophobacter* in *δ-proteobacteria* were identified as dominant in promoting hydrogen, carbon dioxide and methane production, which are all anaerobic bacteria[49,50]. In the class of *γ-proteobacteria*, the highest relative abundance of the generaes were *Enterobacteriaceae*, *Tolumonas*, *Pasteurellaceae*, *Pseudomonas* and *Acinetobacter*. The genus of *pseudomonas* (0.4%) belonging to PAOs is well-associated with the process of phosphorus bio-absorption process occurring in sludge [51,52]. It has been reported that *Enterobacteriaceae* (1.68%) and *Clostridium* (0.2%) were related to the removal of phosphorus by producing phosphine (PH_3_)[53]. Therefore, the production of PH_3_ could be identified as one of the pathways for phosphorus removal. Some scholars considered that *Acinetobacter* and *Pasteurellaceae* belonging to PAOs could use NO_3_^-^, NO_2_^-^ and O_2_ as the electron acceptor to promote phosphorus adsorption in the limited oxygen environment, thus promoting denitrification[54]. The generaes of *Nitrosomonas*(2.41%), *Nitrososphaera*(0.8%) and *Nitrosospira* (0.31%) were all identified as the populations related to nitrosation, and their main function was to achieve nitrosation of ammonia nitrogen[55]. Only 0.01% of the *Nitrospira* belonged to the genus nitrobacteria, resulting in the relatively low concentration of NO_3_^-^ in the reactor, which might be associated with the limited-oxygen condition (below 0.2mg/L) [20,55]. Combined with the analysis of genes and the NO_3_^-^ and NO_2_^-^concentration, the nitrogen (N) is proven to be removed via the SCND process in the OLMBR. Accordingly, the system exhibited good performance on controlling the ammoxidation at the nitrosation stage.

As shown in Fig 10B, other bacteria including *Prevotella*, *Prolixibacteraceae* and *Sphingobacterium* belonging to the class of *Bacteroidetes* were also identified. The main function of *Prolixibacteraceae* and *Sphingobacterium* was to produce organic acid by degrading organic matters [56]. *Caldilinea and Anaerolinea*, belonging to *Chloroflexi*, has the function of degrading carbohydrates in methane producing organisms[57]. *Euryarchaeota* and *Candidatus* are well-associated with the process of denitrifying anaerobic methane oxidation (DAMO) [58,59]. The bioassay results confirm the existence of the DAMO process in the OLMBR. After domestication, their abundance were up to 7.24% and 3.57%. According to the reverse methanogenesis coupling denitrification proposed by Raghoebarsing et al., they could oxidize methane and provide electrons to denitrifying bacteria for the denitrification, especially under the condition of circulating methane aeration[29]. The detection of *Euryarchaeota* and *Candidatus* verifies that our inference on the denitrifying mechanism is correct. At the level of genuses, the highest abundant were *Methanothrix*, *Methanobacterium*, *Methanolinea* and *Methanoregul*, which were mainly responsible for the methane production[60]. Thus, the enriched functional bacteria in the sludge of OLMBR could facilitate multiple reactions including methane production, ammonia oxidation, aerobic nitrosation, denitrification and phosphorus reduction.

### Analysis of mass balance of carbon and nutrients

In order to approve the analysis of microbial community and identify the removal ways of pollutants, the OLMBR reactor was operated for 24 days (see the operating parameters shown in S2 Table in the supporting information). The produced gases were daily evacuated and analyzed using a gas metering pump and a GC; meanwhile, the carbon, nitrogen and phosphorous elements in the increased sludge after 24 days of operation were also analyzed. The results of the mass balance analysis of C, N and P elements are given in Table 1.

**Table 1:** Mass balance of the C, N and P elements in the OLMBR (24 days of operation).

Element	Influent (g)	Effluent		Mass change in emitted Gas (ΔM_g_)		Mass change in sludge (ΔM_S_)		Element recovery
		M_out_ (g)	%	ΔM_g_(g)	%	ΔM_S_ (g)	%	
C	2252.5	101.4	4.5	1521.5(CH_4_)	67.6	219.7	9.9	98.8%
				400.4(CO_2_)	17.8			
N	182.0	17.2	9.7	108.1(N_2_)	61.0	51.9	29.3	97.3%
P	15.6	2.71	18.1	2.9(PH_3_)	19.4	9.3	62.2	95.8%

In regard to the carbon element, the production of CH_4_ (**Δ**M_g_(CH_4_)) after 24 days of operation was 1521.5 g, which indicated that the removal of organic carbon accounted for 67.6%. The mass balance result is consistent with the genetic observation that methanogen was the primary one. The oxidation of carbonaceous substances to carbon dioxide contributed to the 17.8% of the carbon removal. The conversion of the carbon from wastewater into sludge was 219.7 g (9.9%), and this part of carbon was mainly used for the synthesis and growth of cells of microorganisms. The rest of carbon (101.4 g) flowed out from the reactor, and a COD removal efficiency approximate 95.5% was achieved. Compared to the conventional aerobic activated sludge process, the OLMBR produced much less sludge due to the methane production process, alleviating the problem of sludge disposal. The less production of sludge also means that the clean of hollow fiber membrane can be operated with a longer time interval.

In the case of nitrogen, corresponding to the presence of large numbers of denitrifying bacteria in microbial genetic tests, about 61% of N element (108.1g) were removed in the form of nitrogen gas. The fate of N indicated that denitrification process, which was related to the denitrifying bacteria, was the main route for the removal of nitrogen in the reactor. The analysis results also showed that the transformation of N from wastewater into sludge was 51.9 g, accounting for 29.3% of the total nitrogen input. The material balance of phosphorus suggested that the increment of P in the sludge was up to 9.3 g, accounting for 62.2% of the total input of P. About 19.4% phosphorus was removed by the way of producing phosphine, and the production of PH_3_ could be identified as an important path for phosphorus removal in the OLMBR reactor. The mass balance analysis results basically accord with the observation of microbial colony analyses, and well interpret the removal mechanisms of the multiple pollutants.

## Conclusion

In the present study, the removal of COD, N and P in an OLMBR was systematically investigated. A controlled DO concentration around 0.2 mg/L allowed the reactor to achieve a good performance on the simultaneous removal of the pollutants, especially to the NH_4_^+^-N nutrient. Mass balance and bacterial community analysis indicated that the removal of organic carbon was mainly achieved by the methane production process. Short-cut nitrification-denitrification (SCND) was observed as the primary denitrification process in the OLMBR. In addition, a denitrifying anaerobic methane oxidation process also contributed to denitrification. Bio-absorption by PAOs was the main route of the TP removal, and the production of PH_3_ gas also accounted for 19.4% of TP removal. The optimized removal efficiencies of COD, nitrogen (N), and total phosphorus (TP) were approximately 95.5%, 90.0% and 82.6%, respectively. This study suggested that the limited oxygen environment allowed the occurrence of different biological processes. Meanwhile, the hollow fiber membrane enabled the enrichment of relevant bacteria via its interception effect on sludge, therefore, the OLMBR could be used for simultaneous removal of highly concentrated organic, nitrogen and phosphorus in livestock and poultry breeding wastewater.

## Supporting information

S1 Fig GASTEC PH3 gas detecting tube. (DOCX)

S2 Fig PH3 detection result by quick-measuring detector tubes. (DOC)

S1 Table Composition of the gas sampled from the headspace of the OLMBR system. (DOC)

S2 Table The operating conditions of the OLMBR in the experiment for mass balance analysis. (DOC)
