# Supplementary figures and images for: Simultaneous removal of concentrated organics, nitrogen and phosphorus nutrients by an oxygen-limited membrane bioreactor
Source: PLoS One. 2018 Aug 30;13(8):e0202179. doi: 10.1371/journal.pone.0202179 (PMC6116941; doi:10.1371/journal.pone.0202179)

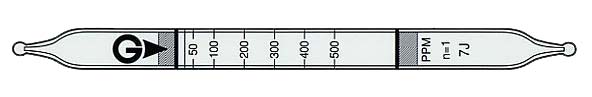


**S1 Fig. GASTEC PH3 gas detecting tube**

Supplement: S1 Fig — (DOCX) [file pone.0202179.s001.docx]
