# Supplementary material for: Simultaneous removal of concentrated organics, nitrogen and phosphorus nutrients by an oxygen-limited membrane bioreactor
Source: PLoS One. 2018 Aug 30;13(8):e0202179. doi: 10.1371/journal.pone.0202179 (PMC6116941; doi:10.1371/journal.pone.0202179)
Supplement: S2 Fig — (DOC) [file pone.0202179.s002.doc]

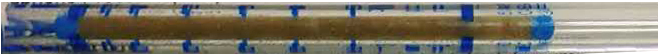


**S2 Fig. PH_3_ detection result by quick-measuring detector tubes**

The agent in the PH_3_ gas detecting tube will react with PH_3_ (2PH_3_ + 6HgCl_2_ + 3H_2_O → Hg_3_P_2_ +3HgCl_2_+3H_2_O+6HCl) and bring about color change that could roughly indicate PH_3_ concentration. In the experiment, the PH_3_ concentration was first detected by GASTEC PH_3_ gas detecting tube and then precisely measured by the Mo-Sb colorimetric method.
