# Supplementary material for: Simultaneous removal of concentrated organics, nitrogen and phosphorus nutrients by an oxygen-limited membrane bioreactor
Source: PLoS One. 2018 Aug 30;13(8):e0202179. doi: 10.1371/journal.pone.0202179 (PMC6116941; doi:10.1371/journal.pone.0202179)
Supplement: S1 Table — (DOC) [file pone.0202179.s003.doc]

**S1 Table. Composition of the gas sampled from the headspace of the OLMBR system.**

| **Constituent** | **CH_4_** | **N_2_** | **CO_2_** | **O_2_** | **H_2_** |
| --- | --- | --- | --- | --- | --- |
| Content (wt %) | 50.7 | 6.9 | 27.9 | 2.7 | 0.06 |
