# Supplementary material for: Simultaneous removal of concentrated organics, nitrogen and phosphorus nutrients by an oxygen-limited membrane bioreactor
Source: PLoS One. 2018 Aug 30;13(8):e0202179. doi: 10.1371/journal.pone.0202179 (PMC6116941; doi:10.1371/journal.pone.0202179)
Supplement: S2 Table — (DOC) [file pone.0202179.s004.doc]

**S2 Table. The operating conditions of the OLMBR** in the experiment for mass balance analysis.

| **Time**  **(d)** | **COD（mg/L）** | | **NH_3_-N（mg/L）** | | **TP（mg/L）** | | **Gas**  **(L)** |
| --- | --- | --- | --- | --- | --- | --- | --- |
|  | **inflow** | **effluent** | **inflow** | **effluent** | **inflow** | **effluent** |  |
| 1 | 4694.0 | 231.3 | 152.9 | 19.6 | 12.78 | 3.14 | 263 |
| 4 | 4670.2 | 179.9 | 151.5 | 17.5 | 12.99 | 2.35 | 212 |
| 6 | 4628.6 | 160.8 | 147 | 18.6 | 12.94 | 1.97 | 210 |
| 8 | 4699.9 | 235.2 | 149.4 | 13.8 | 12.51 | 1.3 | 180 |
| 10 | 4682.1 | 188.5 | 153.7 | 7.8 | 12.78 | 0.54 | 174 |
| 12 | 4563.3 | 187.1 | 153.3 | 13.1 | 13.31 | 2.74 | 242 |
| 14 | 4598.9 | 252.7 | 149.4 | 10.9 | 12.83 | 2.78 | 251 |
| 16 | 4622.7 | 154.33 | 152.5 | 12.8 | 13.69 | 2.43 | 176 |
| 18 | 4640.5 | 182.6 | 154.1 | 18.3 | 12.51 | 3.42 | 256 |
| 20 | 4777.1 | 197.3 | 147 | 14.4 | 13.05 | 1.93 | 220 |
| 22 | 4812.0 | 253.0 | 168.2 | 16.1 | 13.56 | 3.01 | 198 |
| 24 | 4922.27 | 298.1 | 141.2 | 18.4 | 12.98 | 1.56 | 231 |
